# Measurement of Circular Dichroism Spectra without Control of a Phase Modulator using Retardation Domain Analysis

**DOI:** 10.3390/molecules24071418

**Published:** 2019-04-10

**Authors:** Hiroshi Satozono

**Affiliations:** Hamamatsu Phonics K.K, 5000 Hirakuchi, Hamakita-ku, Hamamatsu, Shizuoka 434-8601, Japan; satozono@crl.hpk.co.jp; Tel.: +81-53-586-7111

**Keywords:** circular dichroism, linear dichroism, Mueller matrix, phase modulator, instrument design

## Abstract

This study investigated the measurement of circular dichroism (CD) spectra without controlling a phase modulator. In a conventional CD system, the peak retardation of the phase modulator must remain constant over the observed wavelength range. Thus, the phase modulator must be controlled to maintain an appropriate modulation degree at an observed wavelength. In contrast, CD obtained using retardation domain analysis is not affected by peak retardation. Consequently, CD spectra can be measured without control of the phase modulator, which was experimentally demonstrated in this study. Additionally, linear dichroism spectra were obtained using retardation domain analysis.

## 1. Introduction

Circular dichroism (CD) spectrometers are commonly used for the study of chiral molecules and materials. A conventional method for these spectrometers is the phase modulation technique, which involves a phase modulator and a lock-in amplifier [1]. In this technique, the peak retardation of the phase modulator must remain constant over the observed wavelength range [2,3]. Thus, the modulation degree of the phase modulator must be adjusted to an appropriate degree at an observed wavelength.

In a previous report, we proposed a novel CD measurement method referred to as retardation domain analysis for eliminating artifacts in the CD signal originating from the residual birefringence of the phase modulators [4]. In retardation domain analysis, CD is directly obtained from an element of S02 in the Mueller matrix of a sample (see Appendix A). It should be noted that the peak retardation of the phase modulator does not affect the CD obtained by this analysis. This signifies that the CD spectrum can be obtained without adjusting the modulation degree of the phase modulator. In addition, linear dichroism (LD) of the sample can be simultaneously obtained by retardation domain analysis

In this study, we applied retardation domain analysis to a CD spectrometer and experimentally obtained the CD and LD spectra without controlling the phase modulator. 

## 2. Results and Discussion

### 2.1. Circular Dichroism (CD) Measurement with Various Phase Modulator Settings

**Figure 1:**
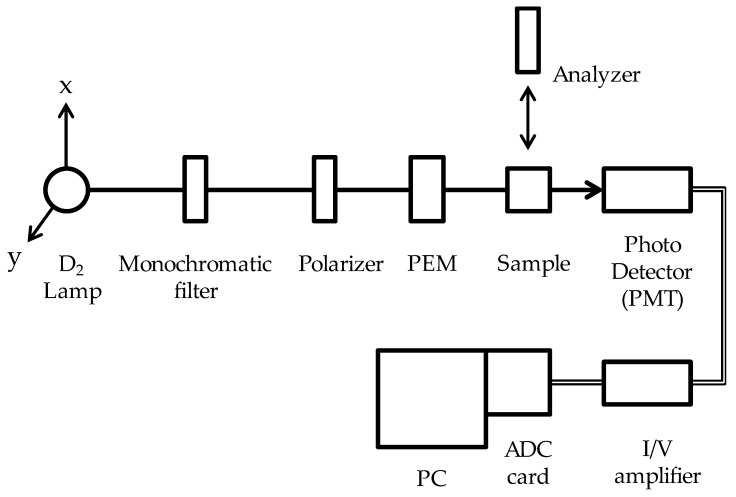
Block diagram of the circular dichroism (CD) measurement system for experiments. (PEM = photoelastic modulator).

Figure 1 presents a diagram of the CD measurement system. The system consists of a light source (D_2_ lamp), a monochromatic filter, a polarizer set along the x-axis, a photoelastic modulator (PEM) rotated 45° from the x-axis to the y-axis, a sample, and a photo detector (photomultiplier tube; PMT). The PEM functions as a phase modulator in this system. The system is virtually identical to a conventional CD measurement system using the phase modulation technique. The sample used consisted of (1*S*)-(+)-10-comphorsulfonic acid ammonium salt (CSA) [5] in distilled water, and the peak transmitted wavelength of the monochromatic filter was 280 nm.

The Stokes vector of light, **I**, detected by the PMT can be calculated using the Mueller matrix method.

**Figure 2:**
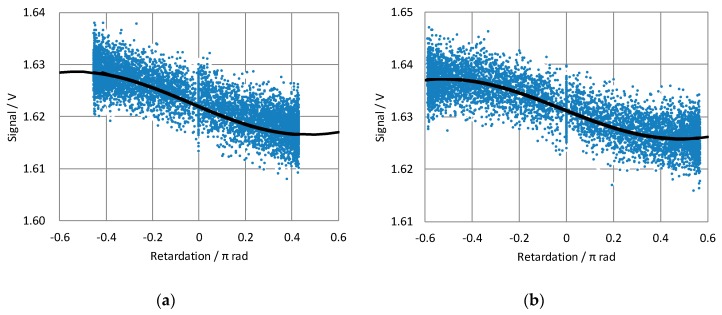
Retardation domain charts of the sample. The peak transmitted wavelength of the monochromatic filter was 280 nm. The peak retardation settings of the photoelastic modulator (PEM) were 0.5π at (**a**) 254 nm and (**b**) 320 nm. The blue dots represent raw data, while the black lines represent the results of curve fitting according to Equation (3).

(1) I=S·M·P·L

where **L** and **I** are the Stokes vectors of the light source and light detected by the PMT. **S**, **M**, and **P** are the Mueller matrices of the sample, the PEM, and the polarizer, respectively. The light intensity of *I*, that is, the first element of **I**, is

(2) $$I\left(\delta \right)=e^{-A}\left(S00+S03\cos\delta -S02\sin\delta \right)$$

where A is the mean absorbance of the sample and *δ* is the retardation of the PEM. S00, S02, and S03 are the element of the Mueller matrix of the sample (see Appendix A for deriving the equation). *I* is a function of the retardation *δ*, that is retardation domain data, *I*(*δ*). The modulation range of the retardation can be set to the wavelength setting of the PEM. The wavelength setting indicates that the peak retardation of the PEM is equal to 0.5π, i.e. the modulation range of the retardation is ±0.5π at the setting wavelength. However, for wavelength settings of 254 nm and 320 nm, the modulation ranges are ±0.454π and ±0.571π at 280 nm, respectively. Figure 2 presents retardation domain data of the sample, in which the wavelength settings of the PEM were 254 nm and 320 nm. The retardation domain data were distributed over the corresponding retardation range. 

Curve fitting for the retardation domain data was performed according to Equation (2). Table 1 presents the fitting results. The retardation domain data was noisy; however, the effect of the noise on S00, S02, and S03 could be reduced because only three variables were determined from many data points.

According to Appendix A, in conventional CD measurement, the CD signal, *I*_CD_, is expressed by

*I*_CD_ = *e*^−*A*^*J*_1_(*δ*_0_)( S03 sin*α* − S02 cos*α*), (3)

where α is the residual static strain birefringence of the phase modulator in Equation (3), a first-order Bessel function, *J*_1_(*δ*_0_), where *δ*_0_ is the peak modulator retardation, is a coefficient of the CD term. Thus, when the wavelength settings of the PEM are 254 nm and 320 nm, the coefficients are 0.547 and 0.581, respectively. The difference in S02 values is much smaller than the difference in coefficients in conventional CD measurement. In this discussion, we assume that S02 is equal to CD, which is reasonable for a solution sample. The results demonstrate that the CD obtained by our analysis is not affected by the wavelength settings of the PEM. The small difference in S02 values is due to the residual effects of noise, as the signal variation is very small and our measurement system lacks a noise reduction system, such as a lock-in amplifier. Additionally, the S03 values corresponding to LDs are zero, which is expected, as the sample is a solution.

### 2.2. Measurement of CD Spectra

**Figure 3:**
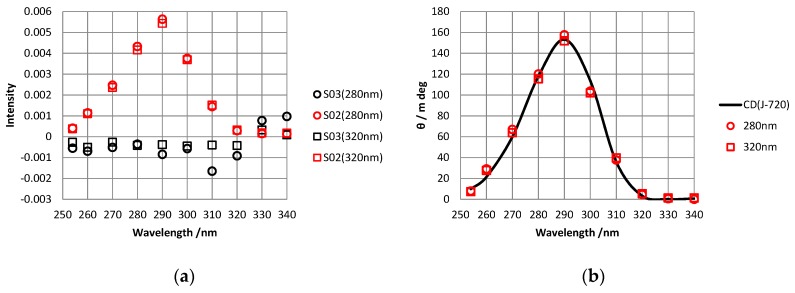
(**a**) Circular dichroism (CD) and linear dichroism (LD) spectra of (1*S*)-(+)-10-comphorsulfonic acid ammonium salt (CSA) in distilled water obtained by retardation domain analysis. We assume that S02 (red) is equal to CD and S03 (black) is equal to LD. The wavelength settings were 280 nm (circles) and 320 nm (squares). (**b**) CD spectrum from a commercial CD spectrometer (black line) and comparison with CD values via retardation domain analysis (red circles and squares).

The CD spectrum of the sample was also measured via retardation domain analysis using the CD measurement system. To obtain the CD at each wavelength, a corresponding monochromatic filter was used. Figure 3a illustrates the obtained CD spectra in which the wavelength settings of the PEM were 280 nm and 320 nm; there is no difference between both CD spectra. Figure 3b presents a comparison of the CD spectra obtained by the retardation domain analysis and a CD spectrum measured by a commercial CD spectrometer. The CD values in Figure 3a were converted to ellipticity in Figure 3b according to Appendix A. As seen in the figure, the CD values obtained by retardation domain analysis are identical to those obtained by the commercial CD spectrometer. 

The standard deviation was calculated by the differences between the CD values obtained by retardation domain analysis and ones by the commercial CD spectrometer to obtain the detection limit of the system used this study. The detection limit at the setting wavelength of 280 nm and 320 nm were both 15.0 mdeg.

The instrumentation used in retardation domain analysis is identical to that used in a conventional CD spectrometer and is thus easy to apply. Our system does not require a lock-in amplifier, which is generally expensive; however, it does require a curve-fitting process. Thus, our method does not enable a real-time measurement, unlike the conventional CD spectrometer. However, this process does not pose a major challenge, as it has become possible to use a high-performance processing device such as a digital signal processor or field-programmable gate array. These devices enable pseudo real-time measurement.

### 2.3. Measurement of Linear Dichroism (LD) Spectra

S03 spectra corresponding to LD spectra can also be obtained simultaneously with CD via retardation domain analysis. Figure 3a presents S03 spectra of the sample in which the wavelength settings of the PEM were 280 nm and 320 nm. The spectra for both wavelengths were mostly zero with several exceptions due to noise. This result is consistent with the fact that the sample was a solution that exhibited no LD.

Measurement of LD is useful for the evaluation of artifacts in CD. The assumption that S02 is equal to CD does not hold if the sample has both LD and linear birefringence (LB). In this case, S02 is the sum of CD and the artifact, which is a combination of LD and LB. Through our analysis, it can be verified that CD does not possess an artifact by determining that the sample exhibits no LD. However, if the sample exhibits large LD, a complex method must be applied to eliminate the artifact [6,7,8,9].

## 3. Materials and Methods 

A D_2_ lamp (L7896, Hamamatsu Photonics, Hamamatsu, Japan), a monochromatic filter set (Asahi bunko, Tokyo, Japan) from 254 nm to 340 nm with 10 nm full width at half maximum (FWHM), a Gran–Taylor prism (GYPB, Sigma Koki, Tokyo, Japan) as a polarizer, a photoelastic modulator (PEM-100 with I/CF50 head, Hinds Instruments, Hillsboro, OR, USA) as a phase modulator, a photomultiplier tube (R1635, Hamamatsu Photonics) as a detector, our own I/V amplifier, and an analog-to-digital converter (LPC-320724, Interface Corp., Hiroshima, Japan) inserted into a PC were used for our CD instrument. (1*S*)-(+)-10-comphorsolfonic acid ammonium salt (CSA, Sigma-Aldrich, Tokyo, Japan) in distilled water without further purification was used as a sample.

Temporal intensity data, *I*(*t*), which were stored in a PC, were converted to retardation domain data by the following method. A second polarizer, called the “analyzer”, was inserted instead of the sample. Temporal data *Ix*(*t*) and *Iy*(*t*) were acquired when the analyzer was set along to x axis and y axis, respectively. The temporal retardation *δ*(t) was calculated by Equation (4) (see Appendix A for deriving the equation). Retardation domain data, *I*(*δ*), was obtained when time *t* in *I*(*t*) was replaced by *δ* in *δ*(*t*).

(4) $$\delta \left(t\right)=\cos^{-1}\left(1-\frac{2\;Iy\left(t\right)}{Ix\left(t\right)+Iy\left(t\right)}\right)$$

*I*(*δ*) was analyzed by our own curve fitting software according to Equation (2) to obtain both S02 and S03 values of the sample. A commercial CD spectrometer (J-720, JASCO, Hachioji, Japan) was also used for evaluating the CD spectra obtained by retardation domain analysis.

## 4. Conclusions

In this study, we have experimentally demonstrated that a CD signal can obtained using retardation domain analysis. The CD obtained from retardation domain data was not affected by the peak retardation of the phase modulator, and CD spectra were obtained with a fixed wavelength setting of the phase modulator. Additionally, S03 spectra related to LD were obtained simultaneously with CD spectra.

There are several advantages to using retardation domain analysis in CD measurement. First, the instrumentation is identical to that used in a conventional CD system; consequently, it can be easily applied to the proposed system. Additionally, a CD spectrum can be obtained without controlling the phase modulator. This feature allows the phase modulator to be easily designed and makes it possible to use an inexpensive modulator. Retardation domain analysis also eliminates artifacts generated by the residual birefringence of the phase modulator [4]. Additionally, LD measurement is useful for evaluating these artifacts.

Large detection limit is a serious problem. The detection limit obtained in this study was worse than that of the commercial CD spectrometer. This originates mainly from the lack of a lock-in amplifier, which is a superior noise reduction system. A low noise detector and amplifier, a noise shield, and a stable light source are required to reduce the noise and to improve the detection limit. Signal averaging is also effective. We are planning to make an improved system to study what determines the detection limit in retardation domain analysis.

## Figures and Tables

**Table 1:** Fitting results for various wavelength settings of the photoelastic modulator (PEM).

	254 nm	320 nm
S00	1.62	1.63
S02	5.95 × 10^−3^	5.80 × 10^−3^
S03	−2.39 × 10^−4^	0.67 × 10^−4^

**Figure A1:**
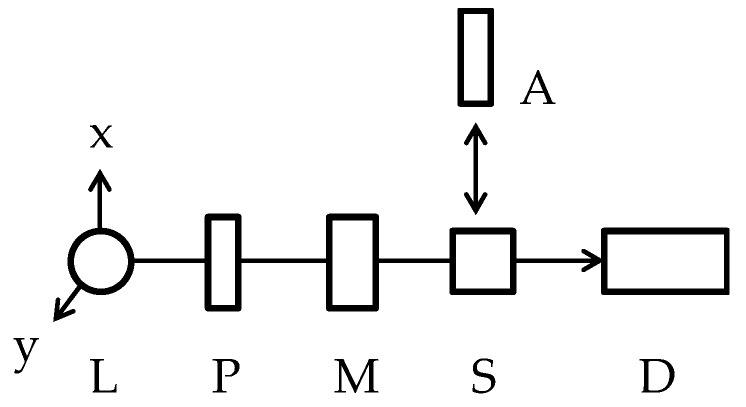
Block diagram of the CD measurement system, which consists of a light source (L), monochromatic filter (F), polarizer (P), phase modulator (M), sample (P), analyzer (A), and photo detector (D).

## Appendix A

Theoretical background of CD measurement by a conventional phase modulation technique and by retardation domain analysis.

Figure A1 illustrates a diagram of the CD measurement system for retardation domain analysis. The system consists of a light source (L), polarizer (P), phase modulator (M), and sample (S), and is identical to a conventional CD spectrometer.

We use the Mueller matrix method to describe the theory of CD measurement by retardation domain analysis, as it is a powerful tool for explaining polarization measurements. In the following discussion, we use notation that includes the Stokes vector and Mueller matrix, which were developed by Go, Jensen, and Shindo [2,4,10,11]. The notation is often used in chemistry but different from the one usually used in physics and optics [12]. To simplify the discussion, the Stokes vector of the light source is entirely non-polarized as follows:

(A1) $$L={\left[\begin{array}{cccc} 1 & 0 & 0 & 0 \end{array}\right]}^{T}$$

The Mueller matrices of the polarizer set along the x-axis, the phase modulator rotated 45° from the x-axis to the y-axis, and a sample with mean absorbance *A* are respectively illustrated below.

(A2) $$P=\frac{1}{2}\left(\begin{array}{cccc} 1 & 0 & 0 & 1\\ 0 & 0 & 0 & 0\\ 0 & 0 & 0 & 0\\ 1 & 0 & 0 & 1 \end{array}\right)$$

(A3) $$M=\frac{1}{2}\left(\begin{array}{cccc} 1 & 0 & 0 & 0\\ 0 & 1 & 0 & 0\\ 0 & 0 & \cos\delta & -\sin\delta \\ 0 & 0 & \sin\delta & \cos\delta \end{array}\right)$$

(A4) $$S=e^{-A}\left(\begin{array}{cccc} S00 & S01 & S02 & S03\\ S10 & S11 & S12 & S13\\ S20 & S21 & S22 & S23\\ S30 & S31 & S32 & S33 \end{array}\right)$$

In the Mueller matrix of the sample, two elements necessary for our discussion are written as follows:

(A5) $$S02=\mathrm{CD}+\frac{1}{2}\left(\mathrm{LB}\prime \mathrm{LD}-\mathrm{LBLD}\prime \right)$$

(A6) S03=LD′sin2θ−LDcos2θ

where LD, LB, and *θ* are the linear dichroism, linear birefringence, and rotation angle of the sample, respectively. LD’ and LB’ are those of the sample rotated 45° from the x axis. The S02 terms, which include both LD and LB, cause significant complications when both LD and LB are large. We therefore assume that the sample is similar to a solution that has relatively small values for LD and LB (<0.1); thus, S02 is approximately equal to CD. The Stokes vector of light detected by the detector is calculated by

(A7) I=S·M·P·L

The resulting light intensity *I* (*δ*) is

(A8) $$I\left(\delta \right)=e^{-A}\left(S00+S03\cos\delta -S02\sin\delta \right)$$

The retardation of the phase modulator is expressed by

(A9) $$\delta =\delta_{0}\sin\omega \;t+\alpha$$

where *δ*_0_ is the peak modulator retardation, ω is the modulation angular frequency, and α is the residual static strain birefringence of the phase modulator. In a conventional modulation method, *I*(*δ*) is analyzed as time domain data, *I*(*t*). The light intensity as a function of time is given by the *n*th Bessel function, *J_n_*.

(A10) $$\begin{array}{ll} I\left(t\right) & =e^{-A}[S00\\ & +S03\left\{-J_{0}\left(\delta_{0}\right)\cos\alpha +2J_{1}\left(\delta_{0}\right)\sin\alpha \sin\omega \;t-\cdots \right\}\\ & -S02\left\{J_{0}\left(\delta_{0}\right)\sin\alpha +2J_{1}\left(\delta_{0}\right)\cos\alpha \sin\omega \;t+\cdots \right\}] \end{array}$$

In the conventional method, the S02 (≈CD) signal can be obtained by extracting the 1ω frequency factor using, for example, a lock-in amplifier. The CD signal, *I*_CD_, is expressed below:

*I*_CD_ = *e*^−*A*^*J*_1_(*δ*_0_)( S03 sin*α* − S02 cos*α*), (A11)

*I*_CD_ is equal to S02 only when α is negligible. Additionally, Equation (A11) implies that the CD intensity is affected by the first-order Bessel function, *J*_1_(*δ*_0_). Thus, the peak retardation of the phase modulator, *δ*_0_, must be adjusted to a constant *J*_1_(*δ*_0_) over the entire wavelength range. In contrast, in retardation domain analysis, the CD signal can be obtained directly from Equation (A8) because S00, S02, and S03 are orthogonal to one another around variable *δ* and can be easily separated. Additionally, there are no factors in the S02 term. The peak retardation of the modulator, *δ*_0_, is only affected over the retardation range in the *I*(*δ*) chart. Therefore, a CD spectrum can be measured in retardation domain analysis without controlling *δ*_0_.

However, the raw data of the sample are time-oriented data, namely *I*(*t*). To convert the data to retardation domain data, namely *I*(*δ*), the time-oriented retardation data of the phase modulator, namely *δ*(*t*), are required. To obtain *δ*(*t*), the following procedure was performed. First, the sample was replaced by a second polarizer referred to as the analyzer (Figure A1). The analyzer was then positioned along the x-axis, and the time-oriented data, *Ix*(*t*), were measured. *Ix*(*t*) is given by the following:

(A12) $$Ix\left(t\right)=\frac{1}{4}\left[1+\cos\delta \left(t\right)\right]$$

Then, the analyzer was positioned along the y-axis, and the time-oriented intensity, *Iy*(*t*), was measured. *Iy*(*t*) became:

(A13) $$Iy\left(t\right)=\frac{1}{4}\left[1-\cos\delta \left(t\right)\right]$$

Hence, *δ*(*t*) was calculated by

(A14) $$\delta \left(t\right)=\cos^{-1}\left(1-\frac{2\;Iy\left(t\right)}{Ix\left(t\right)+Iy\left(t\right)}\right)$$

Unfortunately, the sign of the retardation cannot be obtained by Equation (A14), but it can be determined using several methods, such as the synchronous signal from the phase modulator. Finally, *I*(*δ*) is obtained when time *t* in *I*(*t*) is replaced by *δ* in *δ*(*t*).

Relationship between CD of an element in Mueller matrix and ellipticity.

The definition of CD of an element in Mueller matrix [2] is defined by

(A15) CD=ln10·ΔA/2

And the definition of ellopticity [13] is

(A16) $$\Delta A=\frac{4\pi }{180\cdot \ln10}\theta =\frac{\theta }{32.982}$$

Therefore, the ellipticity can be expressed by CD as follows:

(A17) $$\theta =\frac{180}{2\pi }\mathrm{CD}\;=28.648\cdot \mathrm{CD}$$
